# Moving towards a Healthier Dietary Pattern Free of Ultra-Processed Foods

**DOI:** 10.3390/nu14010118

**Published:** 2021-12-28

**Authors:** Rosa Casas

**Affiliations:** 1 Department of Internal Medicine, Hospital Clinic, Institut d’Investigació Biomèdica August Pi i Sunyer (IDIBAPS), University of Barcelona, Villarroel, 170, 08036 Barcelona, Spain; rcasas1@clinic.cat; Tel.: +34-93-2275745; Fax: +34-93-2275758; 2 CIBER 06/03: Fisiopatología de la Obesidad y la Nutrición, Instituto de Salud Carlos III, 28029 Madrid, Spain

In recent years, ultra-processed food (UPF) intake has increased worldwide, representing almost 60% of total dietary intake in several countries such as the USA and the UK, and around 17 to 24% in the Mediterranean countries, such as Spain and Italy, respectively [1]. Thereupon, this increase has been directly associated with global changes in dietary patterns and the increase in obesity and chronic diseases prevalence. For this reason, there is a growing interest in its impact on health and whether its high consumption could be considered as an unhealthy diet descriptor [2]. Currently, it is well documented that high consumption of UPF is associated with a worse cardiometabolic risk profile and a higher risk of cardiovascular (CVD), cerebrovascular disease, cancer, frailty, overweight and obesity, altered renal function, depression, and all-cause mortality [1,3]. In fact, the Moli-Sani study [4] reported that high consumption of UPFs is associated with a 58% increased risk of CVD mortality, 52% higher risk of dying from ischemic heart disease/cerebrovascular causes, and 26% higher risk of all-cause mortality independently of previously known risk factors, including a global assessment of overall diet quality as reflected by adherence to a Mediterranean diet. Similar results were reported in secondary CVD prevention [1].

Indeed, the strong advertising and the aggressive marketing (reduced price for super-size servings) of this type of hyper-palatable and cheap, processed foods, addressed mainly at children and adolescents, as well as the innovation of the food industry and their omnipresence, may partially explain the changes observed in diet [5,6]. Firstly, this type of ultra-processed food technology became dominant in high-income countries; nevertheless, it is nowadays rapidly increasing in lower- and upper-middle-income countries [5]. Additionally, among the main reasons that could explain the appearance and growth of ultra-processed foods intake, social-economic aspects should be highlighted. In urbanized societies, with the increase in female workforce, a desire to reduce the time spent for cooking and processing food developed, which, along with food environment factors and globalization worldwide, has favored the production and consumption of “ready-to-eat” and “ready-to-heat” products [7]. 

The general definition of UPF indicates that the final food products are formulated with five or more ingredients. These ingredients are usually cheap, industrial sources of fat, carbohydrates, or several additives used to aromatize, provide flavor, and make food more palatable. These additives are industrially formulated mixtures containing little (if any) whole foods [8]. There is a wide variety of UPFs, and, based on the region, they include pastries (cakes, sweets, and industrial bread), instant soups, carbonated soft drinks, ice creams, breakfast cereals, fatty or salty snacks, margarine, processed fruits and vegetables, baked goods, etc. [2]. UPFs are the major dietary contributors of sodium, saturated and *trans* fats, added sugars, etc., while their nutritional quality is generally low (low fiber, proteins, and micronutrients content) [8]. 

In order to develop and apply food policies to counteract the negative effects of the consumption of UPFs and their impact on health, it is important to identify which factors (including sociodemographic and behavioral) are associated with UPF consumption, as well as the main UPF dietary sources [6,7]. Unhealthy dietary patterns characterized by high consumption of energy-dense foods and low intake of fruits and vegetables, together with socioeconomically disadvantaged conditions (lower educational level or socioeconomic position, or individuals living in disadvantaged areas), are directly associated with an unhealthy nutritional profile and higher risk of chronic disease [6,9]. In this sense, Calixto Andrade et al. [10] examined how socioeconomic characteristics and diet quality vary according to UPF intake, in a cross-sectional nationally representative survey, the Étude Nationale Nutrition Santé, of French adults (2642 participants, aged between 18 to 74 years old). First, the authors reported dietary inequalities among French adults according to UPF consumption and the detrimental effect of its consumption on overall diet quality. Almost one-third of the energy intake of French adults was contributed by UPF intake. In addition, a worse dietary profile (higher energy density, and higher intake of total carbohydrates, free sugar, and total and saturated fat) was associated with UPF consumption, young individuals showing the highest UPFs consumption. Similar results have been reported by Marchese et al. [9] and Magalhães et al. [7], who showed how sociodemographic characteristics (age, gender, educational level, marital status, smoking status, living in disadvantaged areas or with lower socioeconomic position) and diet quality modify the consumption of UPF. There is strong evidence for the association between age and UPF consumption. The highest percentage of energy intake derived from UPF intake is found among the youngest (adolescents and younger adults), while older adults (≥45 years of age) showed the lowest percentage of energy from UPF. This high UPF consumption among the youngest can be explained by their ability to easily accept new eating habits and food products, their misconception that healthy food is expensive, and the difficulty of meal planning and/or food shopping during long work or study hours. Moreover, their meal pattern is mainly irregular, and they tend to eat more snacks, fast food, and energy-dense food, compared with older individuals [11,12]. Nevertheless, the data reported by Calixto Andrade et al. [10] showed that UPF consumption is inversely associated with age, Marchese et al. [9] found that elderly individuals (≥71 years) consumed more UPF, compared with adults (51–70 years old). Authors argued that the lack of motivation or access to convenience products may explain the differences observed with other studied populations.

According to Calixto Andrade et al. [10], the dietary contribution of UPF to total energy intake in France (31.1% of daily energy intake) is lower, compared with other countries, due to the preservation of the French traditional culinary culture and the resistance to “westernization” by its population. The industrialization of food systems, technological change, globalization, and inadequate health policies to promote healthier dietary habits lead to a higher supply of UPFs (higher production and wider product variety) and, consequently, increased UPF consumption.

Second, Calixto Andrade et al. [10] found that those individuals who lived in urban areas or had lower educational status (primary school), showed the highest consumption of UPFs; such associations were previously observed in both developed and developing countries. These findings are aligned with the data reported by Marron-Ponce et al. [13], who found that UPF consumption showed an inverse association with age, as well as a direct association with residence in urban regions, such as living in northern Mexico, where sociodemographic factors directly associated with higher intake of UPFs. Similar findings have been reported in Colombia [14].

Third, another study [10] showed the role of educational level on UPF consumption, where individuals with lower educational levels showed a higher intake of UPFs. The lack of nutritional education, together with marketing strategies and the reduced prices of UPFs, easily mislead consumers, promoting its consumption. These findings are consistent with previous results about the inverse association between educational level and UPF consumption [7,9,10]. Nevertheless, studies conducted in Brazil, Colombia, and Mexico reported a higher UPF intake among the richest individuals with higher educational levels [13,15,16]. In contrast, the consumption of UPFs in the UK, France, or the USA was higher among those individuals with lower educational and income levels.

Finally, for future research, it would be of interest to include other socioeconomic, and sociodemographic factors, as well as psychosocial factors such as employment status, family income level, number of children in the household, the educational level and occupation of the head of household, sex, ethnicity, or toxic habits (including smoking status and alcohol intake), in order to identify the most successful health policies and programs to reduce UPF consumption and, consequently, improve diet quality. Considering the robust scientific literature associating UPF consumption with several adverse health outcomes, the implementation of realistic public policies to limit their consumption are necessary. Therefore, developing the desire for healthier lifestyle habits through educational programs that promote healthier food environments, as well as reducing obesogenic food advertising, should be addressed to individuals in all sociodemographic and socioeconomic categories. In this sense, some countries have just started to implement some measures such as taxes on UPF foods, restricting the places suitable for its sale, or advertisement regulations, especially for those advertisements addressed to children or adolescents, in order to discourage their consumption.
